# A Comparative Study of Hip Arthroplasty and Closed Reduction Proximal Femur Nail in the Treatment of Elderly Patients with Hip Fractures

**DOI:** 10.3389/fsurg.2022.904928

**Published:** 2022-05-18

**Authors:** Xianchao Zhou, Xiang Shen

**Affiliations:** First Department of Orthopedics, The Fourth Hospital of Changsha, Changsha, China

**Keywords:** hip fracture, hip arthroplasty, closed reduction, intramedullary nailing, proximal femur nail

## Abstract

**Objective:**

To compare the clinical effect of hip arthroplasty and closed reduction intramedullary nailing of proximal femur in the treatment of elderly hip fracture patients.

**Methods:**

There are 90 elderly hip fracture patients being recruited in the present study. Fifty patients in Group A received closed reduction intramedullary nailing of proximal femur, and 40 patients in Group B received hip arthroplasty. All patients were followed up for 12 months after surgery, clinical outcomes included surgical indicators, visual analog scale (VAS) score, Harris score, quality of life, mental status, and complications.

**Results:**

The surgery time, bleeding volume, infusion volume of patients in Group A are all significantly lower than those in Group B (*p* < 0.05), while the weight-bearing activity time and first workout time of Group A are all significantly higher than those in Group B (*p* < 0.05). The VAS score in patients of Group A at 1 week postoperative is significantly lower than that in patients of Group B (*p* < 0.05). The Harris score in patients of Group A at 3, 6, and 12 months postoperative are all significantly higher than those in patients of Group B (*p* < 0.05), and the excellent and good rate of hip function recovery at 12 months postoperative in patient of Group A is significantly lower than that in patients of Group B (80% vs. 95%, *p* < 0.05). Furthermore, The score of SF-36 standardized physical component, SF-36 standardized mental component and Barthel in patients of Group A at 6 months postoperative are significantly lower than those in patients of Group B (*p* < 0.05), and the score of mini-mental state examination is significantly higher (*p* < 0.05), while there are not significantly different at 12 months postoperative (*p* > 0.05). The incidence of postoperative complications in Group A was significantly lower than that in Group B (10% vs. 27.5%, *p* < 0.05).

**Conclusion:**

Elderly hip fracture patients treated with closed reduction intramedullary nailing of proximal femur has less surgical trauma and lower complication rates, but slower postoperative recovery compared with hip arthroplasty.

## Introduction

Hip fractures are the most common type of fractures in orthopedics, including intertrochanteric fractures, femoral–tibial fractures, and subtrochanteric fractures, with the highest incidence in elderly patients which is due to osteopenia or osteoporosis (1, 2) Hip fractures not only increase mortality and reduce the quality of life in older patients, but also cause patients to be unable to return to their pre-injury living environment, require higher levels of care, and so on (3, 4). Treatment protocols for hip fractures include both non-surgical and operative treatments, but non-surgical treatments have significant disadvantages including fracture malunion, nonunion, and complications from prolonged bed rest (5–7). Therefore, surgery is recommended for all elderly patients with hip fractures who tolerate surgery. Protocols for surgical treatment of hip fractures include dynamic hip screw, dynamic condylar screw, proximal femoral nail, proximal femoral nail antirotation, hip arthroplasty, etc (8).

Currently, proximal femur nail and hip arthroplasty are widely used in the treatment of hip fractures due to their suitability for various types of hip fractures, simple surgery, low blood loss, and low trauma (9, 10). However, many clinical studies have compared the efficacy of proximal femur nail and hip arthroplasty in the treatment of hip fractures, but the results have been inconsistent or even opposite (11, 12). In the present study, we designed to study the clinical effect of hip arthroplasty and closed reduction intramedullary nailing of proximal femur in the treatment of elderly hip fracture patients.

## Patients and Methods

### Patients and Ethics Statement

There were 90 elderly hip fracture patients from January to December 2020 being recruited in the present study. Diagnostic reference for hip fractures “Guideline for the management of hip fractures 2020: Guideline by the Association of Anaesthetists” (13). According to the different surgical treatment methods, they were divided into Group A and Group B. Fifty patients in Group A received closed reduction intramedullary nailing of proximal femur, and 40 patients in Group B received hip arthroplasty. All patients recruited in this study were informed about the details of this study and signed informed consent. In addition, all patients enrolled in this study must meet the following criteria: Inclusion criteria: (1) over 60 years old; (2) hip fracture diagnosed by imaging; (3) surgery performed within 1 week of fracture; and (4) compliance with surgical criteria and signed informed consent. Exclusion criteria: (1) multiple fractures, or surgery performed over 1 week of fracture; (2) deep vein thrombosis, infection, cardiovascular and cerebrovascular disease, malignant tumor; (3) mental disability, intellectual disability, or communication difficulties; (4) Refracture after surgery; (5) failure to complete 12-month follow-up after surgery; (6) failure to cooperate with training; and (7) abnormal functions of organs such as liver and kidney.

### Data Collection

We collected the demographic of patients including age, gender, height, and weight, and recorded the surgery information including American Society of Anesthesiologists (ASA) grade, the surgery time, bleeding volume, infusion volume, and we also recorded hospital stay time, weight-bearing activity time, and first workout time. Moreover, we used visual analog scale (VAS) score to evaluate the pain of patients at 1 h, 24 h, 48 h, and 1 week postoperative.

### Follow-Up

All patients completed a 12-month follow-up after surgery, and the follow-up protocol was as follows: All patients returned to the hospital for imaging examinations and hip function assessments at 3, 6, and 12 months after surgery. And we used the Harris score to evaluate the hip function of patients before treatment and at 3, 6, and 12 months postoperative. According to the Harris score, the hip function recovery of elderly hip fractures patients was rated as excellent (Harris score ≥90), good (80≤ Harris score <90), medium (70≤ Harris score <80), and poor (Harris score <70) (14, 15). At 6 and 12 months postoperative, we used the health survey summary (MOS) item short form health survey (SF-36) and Barthel index to evaluate the life quality of patients, and used mini-mental state examination (MMSE) evaluate the mental state of patients.

### Statistical Analysis

Data in this study were analyzed by SPSS20.0 (National Institute of Health in the USA). We used Student’s *t*-test to compare the differences in measurement data between the two groups, and used the *χ*^2^ test to compare differences in count data. *p* < 0.05 indicated significant difference.

## Results

### Demographic and Perioperative Data

As shown in Table 1, the demographic of patients in Group A including age, gender, height, and weight are all no significantly different with patients in Group B (*p* > 0.05). At the same time, there is also no significant difference in ASA grade and the time of hospital stay between Group A and Group B (*p* > 0.05). However, the surgery time, bleeding volume, and infusion volume of patients in Group A are all significantly lower than those in Group B (*p* < 0.05), while the weight-bearing activity time and first workout time of patients in Group A are all significantly higher than those in Group B (*p* < 0.05) (Table 1).

**Table 1:** Comparison of demographic and perioperative data between the two groups.

Variables	Group A (*n* = 50)	Group B (*n* = 40)	*χ*^2^/*t*	*p*-value
Age (years)	68.8 ± 2.3	69.0 ± 2.1	0.186	0.860
Gender (male/female, *n*)	23/27	18/22	0.009	0.925
Height (cm)	165.2 ± 10.2	164.8 ± 10.9	0.358	0.445
Weight (kg)	63.5 ± 7.8	63.9 ± 7.2	0.863	0.235
ASA (I + II/III, *n*)	42/8	31/9	0.613	0.434
Surgery time (min)	72.8 ± 10.1	89.5 ± 13.8	6.382	<0.001
Bleeding volume (ml)	234.8 ± 43.5	297.8 ± 56.7	7.013	<0.001
Infusion volume (ml)	115.8 ± 23.9	142.3 ± 35.9	6.265	<0.001
Hospital stay time (day)	15.8 ± 3.5	14.7 ± 3.8	0.627	0.279
Weight-bearing activity time (day)	4.3 ± 0.8	3.2 ± 0.6	8.126	<0.001
First workout time (day)	18.9 ± 3.8	12.3 ± 2.5	20.358	<0.001

### Postoperative Pain

There is no significant difference in VAS score at 1, 24, and 48 h postoperative between Group A and Group B (*p* > 0.05), while the VAS score in patients of Group A at 1-week postoperative is significantly lower than that in patients of Group B (*p* < 0.05) (Table 2).

**Table 2:** Comparison of VAS scores at different time after operation between two groups of patients (score, $\bar{x}\pm S$).

Group	*n*	1 h	24 h	48 h	1 week
Group A	50	0.9 ± 0.2	4.2 ± 0.6	4.6 ± 0.8	1.2 ± 0.2
Group B	40	0.9 ± 0.3	4.3 ± 0.7	4.8 ± 0.9	2.5 ± 0.4
*t*		0.023	0.493	0.981	2.568
*p*		0.838	0.463	0.129	0.039

### Hip Function Recovery

There is no significant difference in Harris score between Group A and Group B preoperative (*p* > 0.05), while the Harris score in patients of Group A at 3, 6, and 12 months postoperative are all significantly higher than those in patients of Group B (*p* < 0.05) (Table 3). Furthermore, according to the Harris score at 12 months postoperative, the number of patients in Group A whose hip function was rated as excellent, good, medium, and poor are 15, 25, 8, and 2, respectively. And Group B are 23, 15, 2, and 0, respectively. Importantly, the excellent and good rate of hip function recovery at 12 months postoperative in patient of Group A is significantly lower than that in patients of Group B (80.0% vs. 95.0%, *p* < 0.05) (Table 4).

**Table 3:** Comparison of Harris score between two groups of patients at different time after operation (score, $\bar{x}\pm S$).

Group	*n*	Preoperative	Postoperative (months)		
			3	6	12
Group A	50	46.2 ± 5.9	68.2 ± 7.2	79.6 ± 11.2	92.5 ± 10.8
Group B	40	46.9 ± 6.3	60.9 ± 10.2	68.5 ± 12.8	85.7 ± 11.6
*t*		1.059	13.826	19.213	6.894
*p*		0.531	<0.001	<0.001	<0.001

**Table 4:** Comparison of hip joint function recovery between two groups of patients [*n* (%)].

Group	*n*	Excellent	Good	Medium	Poor	Rate of excellent and good
Group A	50	15 (30.0)	25 (50.0)	8 (16.0)	2 (4.0)	40 (80.0)
Group B	40	23 (57.5)	15 (37.5)	2 (5.0)	0 (0.0)	38 (95.0)
*t*						4.327
*p*						0.038

### Other Clinical Outcomes

The score of SF-36 (PCS), SF-36 (MCS), and Barthel in patients of Group A at 6 months postoperative are significantly lower than those in patients of Group B, while the score of MMSE in patients of Group A at 6 months postoperative is significantly lower than that patients of Group B (*p* < 0.05) (Table 5). At 12 months postoperative, there are no significance in the score of SF-36 (PCS), SF-36 (MCS), Barthel, and MMSE between Group A and Group B (*p* > 0.05) (Table 6).

**Table 5:** Comparison of life quality and mental state at 6 months postoperative between two group (score, $\bar{x}\pm S$).

Group	*n*	SF-36 (PCS)	SF-36 (MCS)	Barthel	MMSE
Group A	50	35.1 ± 6.8	50.7 ± 7.5	20.5 ± 1.5	83.2 ± 8.3
Group B	40	40.3 ± 6.5	59.2 ± 6.7	26.3 ± 2.4	76.3 ± 8.8
*t*		9.562	10.319	8.128	1.319
*p*		<0.001	<0.001	<0.001	0.325

*SF-36, the MOS item short form health survey; PCS, standardized physical component; MCS, standardized mental component; MMSE, mini-mental state examination.*

**Table 6:** Comparison of life quality and mental state at 12 months postoperative between two group (score, $\bar{x}\pm S$).

Group	*n*	SF-36 (PCS)	SF-36 (MCS)	Barthel	MMSE
Group A	50	30.3 ± 4.2	42.7 ± 6.1	17.5 ± 1.1	87.4 ± 6.1
Group B	40	31.4 ± 3.9	43.8 ± 5.3	18.2 ± 1.8	85.9 ± 7.2
*t*		0.912	1.381	0.521	0.289
*p*		0.139	0.087	0.392	0.169

*SF-36, the MOS item short form health survey; PCS, standardized physical component; MCS, standardized mental component; MMSE, mini-mental state examination.*

### Postoperative Complications

All patients were followed up for 12 months after surgery, there were 1 infection, 3 deep vein thrombosis (DVT), and 1 bedsore in Group A, while 1 infection, 2 built-in loose, 2 DVT, 2 bedsores, and 4 cardiovascular disease occurred in Group B. Namely, the incidence of postoperative complications in Group A was significantly lower than that in Group B (10% vs. 27.5%, *p* < 0.05) (Table 7).

**Table 7:** Comparison of postoperative complications between the two groups of patients [*n* (%)].

Group	*n*	Infection	Built-in loose	DVT	Bedsores	Cardiovascular diseases	Total rate
Group A	50	1 (2.0)	0 (0.0)	3 (6.0)	1 (2.5)	0 (0.0)	5 (10.0)
Group B	40	1 (2.5)	2 (5.0)	2 (5.0)	2 (5.0)	4 (10.0)	11 (27.5)
*t*							4.656
*p*							0.031

*DVT, deep vein thrombosis.*

## Discussion

Hip fracture is the most common fracture in the elderly, accounting for about 3%–4% of the whole-body fracture, which not only seriously affects the daily life of patients, but also increases the risk of death of patients (1, 2). At present, non-surgical conservative treatment, internal fixation surgery, and artificial hip replacement are clinically used for the treatment of patients with different types of hip fractures. Among them, non-surgical conservative treatment is only recommended for patients who cannot tolerate surgical treatment due to its long treatment cycle, poor treatment effect, and long-term bed rest. Surgery is currently the preferred treatment protocol recommended for hip fracture patients (5–7). Internal fixation and artificial hip arthroplasty are the main surgical methods for hip fracture surgical treatment, and the advantages are not only reduced trauma and shortened bed time, but also significantly reduced the incidence of complications, promoted functional recovery of the hip joint and improved the life quality of hip fracture patients (9, 10).

In this study, elderly hip fracture patients received hip arthroplasty or closed reduction proximal femur nail treatment, and we found that compared with hip arthroplasty, closed reduction proximal femur nail treatment had less surgery time, bleeding volume, infusion volume, but higher weight-bearing activity time and first workout time, which suggested closed reduction proximal femur nail treatment is less traumatic for elderly fracture patients, while hip arthroplasty treatment patients recover faster after surgery. In addition, we also found that the postoperative long-term hip function recovery of hip arthroplasty patients was better than closed reduction proximal femur nail treatment, but there was no significant difference in postoperative long-term quality of life and mental state between the two groups.

Artificial hip arthroplasty is the most commonly used surgical treatment in clinical practice, and its advantages are that the postoperative joint mobility is good, the stability is high, and the patient can get out of bed early without waiting for the fracture site to heal (16, 17). Bone cement-type and bio-type artificial hip joints are two groups of prostheses commonly used in the clinic, and they have different characteristics when used in the treatment of elderly patients with hip fractures (18, 19). Bone cement artificial hip joint can improve early stability, help get out of bed early, and promote recovery, but different degrees of acute hypotension, hypoxemia, arrhythmia, cardiac arrest, and cardiopulmonary dysfunction may occur (20, 21). The initial stability of the biological artificial hip joint is not ideal, and long-term bed rest is required after the operation (21). Therefore, the effect of short-term follow-up after the operation is not as good as that of the cemented artificial hip joint, but the risk of postoperative unsafe events is relatively low. In the present study, since the subjects included in this study were all elderly patients with hip fracture, osteoporosis, and severe bone loss were common, so a cemented artificial hip joint was selected during the operation to improve the early stability. Proximal femoral nail fixation is a minimally invasive intramedullary fixation procedure based on biomechanical principles using an anti-rotation helical blade to fix the femoral neck. Because the helical blade is very close to the bone, it can prevent fracture rotation and varus deformity, and enhance fracture stability (22–25). As a minimally invasive operation, small surgical incision, small trauma, short operation time, and avoiding periosteum and soft tissue dissection are the inherent advantages of proximal femoral nailing (26, 27).

Postoperative complications are the main risk factors affecting the recovery of elderly patients with hip fracture (28, 29). Bone cement poisoning, including cardiovascular disease, pulmonary dysfunction, and hypotension, is the main postoperative complication of hip arthroplasty (20, 21). In this study, there were 11 postoperative complications in elderly hip fracture patients treated with hip arthroplasty, among which the highest incidence of cardiovascular disease (four cases). Compared with hip replacement, the postoperative complication rate of elderly hip fracture patients treated with proximal femoral nail fixation is lower, only 10%. This is mainly due to the low trauma to patients treated with proximal femoral nail fixation. However, it should be noted that although the postoperative complication rate of patients treated with proximal femoral nail fixation was significantly lower than that treated with hip arthroplasty, patients treated with hip arthroplasty had faster recovery of hip function. This is mainly due to the timely intervention of postoperative complications to reduce their impact on patients’ postoperative recovery.

## Conclusion

Hip arthroplasty and proximal femoral nail fixation have different advantages for elderly patients with hip fractures. The advantages of proximal femoral nail fixation are ease of operation, less trauma, less operative time, and postoperative complication rates, while the advantages of hip arthroplasty are faster postoperative recovery. Therefore, for different elderly hip fracture patients, different surgical treatment methods should be selected according to their actual status.

## Data Availability

The original contributions presented in the study are included in the article/Supplementary Material, further inquiries can be directed to the corresponding author/s.
